# Analyse des facteurs prédictifs de malignité des goitres nodulaires : à propos de 500 cas

**DOI:** 10.11604/pamj.2016.23.88.8405

**Published:** 2016-03-15

**Authors:** Brahim Bouaity, Youssef Darouassi, Mehdi Chihani, Mohamed Mliha Touati, Haddou Ammar

**Affiliations:** 1 Service d'Oto-rhino-laryngologie, Hôpital Militaire Avicenne, Marrakech, Maroc

**Keywords:** Goitre nodulaire, malignité, échographie, Nodular Goiter, malignancy, ultrasound

## Abstract

Les nodules thyroïdiens sont très fréquents et moins de 10% d'entre eux sont malin. Ils posent un véritable problème diagnostique et thérapeutique surtout par rapport à leur nature bénigne ou maligne. L’étude de certains facteurs cliniques et paracliniques de présomption de malignité permet de bien codifier la stratégie thérapeutique. Le but de ce travail est d’étudier les facteurs prédictifs de malignité des goitres nodulaires et comparer nos résultats à ceux de la littérature. Il s'agit d'une étude rétrospective à propos de 500 cas de goitres nodulaires opérés au service d'Oto-rhino-laryngologie (ORL) et Chirurgie cervico-faciale (CCF) de l'hôpital militaire Avicenne de Marrakech entre 2006 et 2012. Le pourcentage de cancers a été de 6,8%. L’âge moyen de nos patients était de 46 ans, avec une sex-ratio de 5 (F/H). A la palpation cervicale; le caractère dure du nodule a été constaté dans 94,4% des cas de cancer, avec des limites irrégulières dans 64,70% des cas de cancer. Trois nodules étaient fixes et ils étaient tous malins. Les adénopathies cervicales ont été constatées chez 8 malades dont 7 présentaient des cancers. A l’échographie, 61,8% des nodules malins présentaient un aspect hypoéchogène, avec des contours flous dans 88,24% des cas. La vascularisation intra nodulaire était présente dans 35,3% de ces cas des cancers avec des microcalcifications chez 55,9% d'entre eux. Le halo hypoéchogene périnodulaire était incomplet dans 73,5% des cas de cancer. Nos patients étaient en euthyroïdie dans 84,6% des cas. Les facteurs prédictifs de malignité d'un goitre nodulaire, étaient donc dans notre étude d'abord cliniques: l’âge supérieur à 60 ans, la consistance dure du nodule, sa fixité, son caractère irrégulier et mal limité à la palpation, ainsi que la présence d'adénopathie(s) cervicale(s) à l'examen; et échographiques: le caractère hypoéchogène, les limites floues, la présence de microcalcifications et la visualisation d'une vascularisation intranodulaire avec ou sans vascularisation périnodulaire. Bien que certains de ces facteurs soient fortement prédictifs de malignité, seule l'histologie définitive apporte le diagnostic de certitude. Le clinicien doit alors se baser sur un faisceau d'arguments pour adopter une conduite pratique en vue d'une prise en charge adéquate.

## Abstract (en)

Thyroid nodules are very common and less than 10% of them are malignant. They pose a serious diagnostic and therapeutic problem with respect to their benign or malignant nature. The study of some clinical and paraclinical factors for presumed malignancy makes it possible to codify appropriate therapeutic strategy. The aim of this study was to investigate predictors of malignancy in nodular goiters and to compare our results with those reported in the literature. This retrospective study consisted of 500 cases of nodular goiter operated in the Department of Otorhinolaryngology (ear, nose and throat or ENT) and Head and Neck Surgery at Avicenne military hospital in Marrakech between 2006 and 2012. The percentage of cancers was 6,8%. The average age of our patients was 46 years, with a sex-ratio of 5 (F/H). The hard nature of the nodule was present in 94,4% of the cases of cancer by palpation; irregular boundaries were present in 64.70% of the cases of cancer. Three nodules were fixed and malignant. Cervical lymphadenopathy were observed in 8 patients, 7 of which had cancer. Ultrasound examination showed hypoechoic appearance in 61,8% of the malignant nodules, with smooth-edges in 88,24% of the cases. Intranodular vascularization was found in 35,3% of the cases of cancers, with microcalcifications in 55,9% of the cases. Perinodular hypoechoic halo was incomplete in 73,5% of the cases of cancer. Our patients were euthyroid in 84,6% of the cases. Predictors of malignancy in nodular goiters were present in our first clinical study: patients over the age of 60 years, hard consistency of nodule, fixity, irregular and poorly defined character by palpation, as well as presence of cervical adenopathy on examination; and echographic features: hypoechoic character, smooth-edges, presence of microcalcifications and visualization of intranodular vascularization with or without peri-nodular vascularization. Although some of these factors are highly predictive of malignancy, only final histology provides certainty of diagnosis. Clinicians should base their clinical practice and patient management on a body of clinical arguments.

## Introduction

La pathologie thyroïdienne comporte une grande variété d'affections de natures différentes par leurs caractéristiques fonctionnelles et anatomopathologiques, dont le goitre nodulaire représente le motif de consultation le plus fréquent. Dans la grande majorité des cas, ces nodules sont bénins, moins de 10% d'entre eux sont des cancers [1]. Le but de cette étude est de montrer l'intérêt d'analyser des critères, cliniques et para-cliniques de présomption de malignité des goitres nodulaires afin de sélectionner les nodules suspects de malignité en vue de réduire le nombre des interventions chirurgicales inutiles et par conséquent la morbidité chirurgicale.

## Méthodes

Il s'agit d'une étude rétrospective sur 500 patients opérés pour goitre nodulaire au service d'Oto-Rhino-Laryngologie (ORL) et de Chirurgie Cervico-faciale (CCF) de l'hôpital militaire Avicenne de Marrakech entre janvier 2006 et décembre 2012. Pour chaque patient nous avons étudié les données épidémiologiques (sexe, âge, lieu de résidence), cliniques (antécédents, durée d’évolution, données de l'examen clinique), para cliniques (biologiques, radiologiques, cytologiques,‥) et anatomopathologiques. Ces données ont été traitées au service d’épidémiologie de la faculté de médecine et de pharmacie de Marrakech.

## Résultats

La fréquence de malignité des goitres nodulaires dans notre série est de 6,8%. L’âge moyen de nos patients était de 46 ans (extrêmes 16-81 ans), 44 ans pour les pathologies bénignes et 48 ans pour les cancers thyroïdiens avec 2 pics de fréquence vers 40 ans et vers 70 ans. La prédominance féminine a été notée dans notre série, avec un sex-ratio de 5 F/H pour l'ensemble des patients étudiés. Il a été de 5,74 pour les pathologies bénignes et de 2,09 pour les tumeurs malignes. Toutes les régions du Maroc ont été concernées avec une nette prédominance au niveau des régions montagneuses. L'irradiation cervicale n'a été retrouvée chez aucun de nos patients. Quatorze patients (12,8%) avaient des antécédents de chirurgie thyroïdienne dont 4 ont présenté un nodule malin. La masse basi-cervicale était le motif de consultation le plus fréquent avec un délai moyen de consultation de 44 mois pour la pathologie nodulaire bénigne et de 56 mois pour celle maligne, associée à des signes de compression chez 14 patients (10 cas de dyspnée dont 3 présentaient un nodule malin, 3 cas de dysphonie par paralysie récurrentielle, un seul cas de dysphagie). Tous les nodules fixes étaient des cancers. Les Tableau 1 et Tableau 2 résument la corrélation de la consistance et les limites de la tuméfaction avec la nature histologique. Les adénopathies cervicales ont été retrouvées chez 8 patients dont 7 présentaient un nodule malin. Deux cas de paralysies récurrentielles ont été retrouvé dont un présentait un cancer. 85,3% des goitres nodulaires bénins s'associaient à une euthyroïdie. Le dosage de la thyrocalcitonine réalisé chez un seul patient était normal. La radiographie thoracique avait objectivé une compression trachéale chez 7 patients dont 5 présentaient une pathologie maligne. Quant aux données échographiques, 85,3% des nodules malins présentaient une taille supérieure à 2 cm contre 48,5% de ceux bénins. Les contours flous ont été retrouvés dans 88,2% des cas de nodules malins contre 4,7% de ceux bénins. Le Tableau 3 résume les caractéristiques échographiques des nodules. Le Tableau 4 montre la corrélation entre type histologique et vascularisation des nodules. La scintigraphie réalisée chez seulement 44 patients montrait le caractère froid dans 20,6% des nodules malins contre 4,9% de ceux qui sont bénins. La cytoponction est devenue un examen incontournable dans le dépistage de la malignité des goitres nodulaires mais nous n'avons pas pu conclure de son intérêt vu le faible nombre d'examens réalisés (7 cas).

**Tableau 1:** Corrélation entre la nature histologique et la consistance du nodule

			Lésions bénignes	Lésions malignes	Total
consistance	**dure**	Effectif	1	17	18
		%	5.6	94.4	100.0
	**ferme**	Effectif	440	17	457
		%	96.3	3.7	100.0
	**molle**	Effectif	25	0	25
		%	100.0%	0.0%	100.0%
Total		Effectif	466	34	500
		%	93.2%	6.8%	100.0%

**Tableau 2:** Corrélation entre la nature histologique et les limites des nodules

	limites				Total
	régulières	%	irrégulières	%	
**Goitres nodulaires bénins**	459	98,50	7	1,50	466
**Goitres nodulaires malins**	12	35,30	22	64,70	34
	471	94,2	29	5,8	500

**Tableau 3:** Structures et caractéristiques des nodules en pourcentage

	Lésions bénignes n(%)	Lésions malignes n(%)
Hypoéchogène (n = 261)	240(91,95)	21(8,05)
Anéchogène (n = 62)	60(96,77)	2(3,22)
Hyperéchogène (n = 78)	63(80,76)	15(19,24)
Isoéchogène (n = 136)	130(95,58)	6(4,42)
Hétérogène (n = 327)	301(92,04)	26(7,96)
Présence de calcifications (n = 43)	24(5,9)	19(55,9)

**Tableau 4:** Corrélation entre type histologique et vascularisation intra nodulaire

	Pas de vascularisation		Vascularisation présente		Total
	Nombre	%	Nombre	%	
**Goitres nodulairs bénins**	460	98,71	6	1,29	466
**Goitres nodulairs malins**	22	64,70	12	35,30	34
**Total**	482	96,4	18	3,62	500

## Discussion

Les goitres nodulaires occupent une place importante en pathologie thyroïdienne, et l'identification des facteurs prédictifs de malignité de ces goitres est un enjeu majeur dans leur prise en charge. Nous avons essayé dans cette étude rétrospective d'identifier des critères épidémiologiques, cliniques et paracliniques de présomption de malignité et de comparer nos résultats à ceux de la littérature. Le pourcentage de cancers dans notre série (6,8%) est superposable à celui de la littérature, évalué entre 5,51% et 8,60% [2, 3]. La prédominance féminine est classique en matière de nodules thyroïdiens avec un sex-ratio de 5 dans notre série comparable à celle de la littérature. Le sexe masculin est un élément classique de présomption de malignité [4], mais il n'est pas retrouvé dans notre série. Le risque de malignité est plus important pour les âges extrêmes (inférieur à 20 ans et supérieur à 60 ans) [4], alors que dans notre série nous avons trouvé ce risque élevé après 60 ans. Le cancer de la thyroïde est plus fréquent dans les zones d'endémie goitreuse [3], ce qui est le cas pour la majorité de nos patients qui proviennent des régions montagneuses (zones de carence iodée). Le seul antécédent personnel qui joue classiquement un rôle important dans l'apparition des nodules thyroïdiens (quelle que soit leur nature) est l'exposition aux radiations ionisantes pendant l'enfance [5], avec des proportions rapportées d'environ deux tiers pour les adénomes et un tiers pour les cancers [6]. La relation entre antécédents familiaux et nature histologique reste discutée, certains ont montré que la présence de goitre bénin dans la famille et en faveur de bénignité, alors que le risque de malignité devient important en présence d'antécédents familiaux de cancer thyroïdien [7, 8]. Dans notre série il n'y avait pas de relation significative entre le risque de malignité et les antécédents familiaux de thyropathie. Certaines caractéristiques des nodules sont très évocatrices de malignité. L'ancienneté des nodules ne doit pas, non plus, faire écarter leur potentiel malin, du fait de l’évolution à bas bruit des cancers. Bien que la plupart des auteurs s'accordent à insister sur l'aspect suspect des nodules qui augmentent rapidement de volume [9], leur stabilité ou leur évolution progressive ne doivent pas être rassurants comme cela a été confirmé dans notre série (délai moyen de consultation de 56 mois pour les cancers).

Le goitre multi nodulaire était longtemps considéré «bénin» par rapport au nodule solitaire [10, 11]. Cette notion a été révisée par plusieurs auteurs qui trouvent plutôt que le risque de malignité serait identique pour les deux groupes [11]. Dans notre série ce risque était le même. La dureté du nodule à la palpation est fortement évocatrice de malignité, avec un taux variant de 21 à 64% [4, 7]. Dans notre série 94,4% des cas de cancers présentaient une consistance dure. De même, la fixité et l'irrégularité des contours des nodules orientent fortement vers leur caractère malin [7], avec un risque de 100% dans notre série pour le caractère fixe. La présence d'une dysphonie, d'une dysphagie et/ou d'une dyspnée serait en rapport avec la malignité par compression ou infiltration des organes de voisinage [12]. Dans notre série, le faible nombre de cas de compression ne permet pas de conclure de sa valeur prédictive. Alors que dans notre série comme dans la littérature, la présence l'adénopathie(s) cervicale(s) est très suspecte de malignité. Notre étude confirme les données de la littérature que le statut hormonal thyroïdien n'est pas un facteur prédictif [4, 7]. Un taux élevé de thyrocalcitonine évoque fortement un carcinome médullaire. Sur la radiographie du thorax, seul la découverte de métastases pulmonaires et/ou d'adénopathies médiastinales sont en faveur de la pathologie néoplasique [13]. Pour certains auteurs [11] la taille des nodules est un facteur prédictif de malignité, de même pour notre étude dont 85,3% des cas de cancer présentaient une taille supérieure à 2 cm.

Les nodules hypo-échogènes sont les plus fortement suspects de malignité dans la littérature [11] et dans notre série (61,8% des nodules malins étaient hypo-échogènes). Les contours flous ou irréguliers sont un signe de suspicion de malignité [4, 14], dans notre étude ils étaient retrouvés dans 88,2% des cas de nodule malins contre 4,7% de ceux bénins, alors que le halo hypo-échogène péri-nodulaire était incomplet dans 73,4% des cas de nodules malins, et ces résultats sont conformes donc aux données de la littérature [11]. La présence de calcifications fait évoquer fortement la malignité pour certains auteurs, alors que d'autres prônent le contraire [11]. Dans notre série, 55,9% des nodules malins contenaient des calcifications, contre 8,6% des nodules bénins. La vascularisation intranodulaire associée ou non à une vascularisation périphérique au doppler suspecte fortement la malignité [11, 14], elle a été retrouvée dans 35,30% des cas de nodule malins dans notre série ce qui est concordant avec les données de la littérature de même que la présence d'adénopathies cervicales est un excellent signe prédictif de malignité. L'utilisation de la scintigraphie s'est nettement réduite ces dernières années. La littérature s'aligne sur le fait que la plupart des cancers thyroïdiens sont hypofixants [11], mais la majorité des nodules hypofixants sont bénins. Le caractère hyperfixant ne doit pas être rassurant. Dans notre étude où la scintigraphie n’était pas systématique nous n'avons pas trouvé de relation significative entre la malignité et l'aspect scintigraphique. La cytoponction à l'aiguille fine a une sensibilité allant de 80 à 97% et une spécificité de 70 à 80% [15]. Une réponse «maligne» est un très grand argument de malignité. Inversement, une réponse «bénigne» est intéressante mais non formelle et une réponse «douteuse» est peu informative. Devant le faible nombre de cytoponctions réalisées chez nos patients (7 cas) nous n'avons pas pu conclure de l'intérêt de cet examen dans le la présemption de malignité.

## Conclusion

Le goitre nodulaire représente le motif de consultation le plus fréquent en matière de pathologie thyroïdienne. La difficulté de sa prise en charge est la distinction des nodules bénins où l'on peut se contenter d'une surveillance de ceux malins qu'il faut opérer. D'où l'intérêt d'une analyse clinique et paraclinique pertinente qui reste très contributive dans l'identification pré chirurgicale des facteurs prédictifs de malignité de ces goitres nodulaires en vue d'une meilleure prise en charge.

### Etat des connaissance sur le sujet

- Les nodules thyroïdiens sont très fréquents mais moins de 10% sont malin
- Ils posent un véritable problème diagnostique et thérapeutique par rapport à leur nature bénigne ou maligne.
- L’étude de certains facteurs cliniques et paracliniques de présomption de malignité permet de bien codifier la stratégie thérapeutique.

### Contribution de notre étude à la connaissance

- Le sexe masculin comme élément de présomption de malignité n'est pas retrouvé dans notre série.
- Pas de relation significative entre le risque de malignité et les antécédents familiaux de thyropathie.
- La taille des nodules est un facteur prédictif de malignité.
